# Assessment of the Risk of Suicide-Related Events Induced by Concomitant Use of Antidepressants in Cases of Smoking Cessation Treatment with Varenicline and Assessment of Latent Risk by the Use of Varenicline

**DOI:** 10.1371/journal.pone.0163583

**Published:** 2016-09-22

**Authors:** Hayato Akimoto, Shinji Oshima, Akio Negishi, Kousuke Ohara, Shigeru Ohshima, Naoko Inoue, Daisuke Kobayashi

**Affiliations:** 1 Faculty of Pharmaceutical Sciences, Josai University, Sakado, Saitama, Japan; 2 Faculty of Pharmaceutical Sciences, Josai International University, Togane, Chiba, Japan; University of Rome Tor Vergata, ITALY

## Abstract

Smoking Cessation Treatment (SCT) is a policy that has to be promoted for health economics, and expectations for the success of treatments with varenicline (VAR) are large. However, the Food and Drug Administration (FDA) have issued a warning on VAR-induced depression and suicide. In the present study, utilizing the FDA Adverse Event Reporting System (FAERS), we searched for antidepressants (ADs) used during SCT that cause fewer suicide-related events (SRE) (Study 1). We also investigated whether VAR concomitantly administered with ADs increases the risk of SRE (Study 2). In addition, we investigated whether the use of VAR alone is a latent risk factor of SRE. The backgrounds of cases with and without SRE were matched using the Propensity Score. In Study 1, the highest integrated Reporting Odds Ratio (iROR) was noted in concomitantly administered mirtazapine (iROR 6.98; 95% Confidence Interval (CI) 1.57–30.99), while the lowest ratio was noted in concomitantly administered amitriptyline (iROR 0.59; iROR95%CI 0.23–1.50). Study 2 clarified that SCT increases the risk of SRE in AD-treated cases (iROR 8.02; iROR95%CI 5.47–11.76; not significance). Of ADs concomitantly used during SCT with VAR, amitriptyline and mirtazapine showed the lowest and highest risks, respectively (Study 1). It was clarified that concomitant use of VAR in the treatment of depression with ADs increased the risk of SRE (Study 2). The results of Studies 1 and 2 suggested that the use of VAR alone is a latent risk factor inducing suicide.

## Introduction

Cigarette smoking is a risk factor for various diseases, such as cancers including lung cancer, chronic obstructive pulmonary disease (COPD), hypertension, ischemic heart disease, and cerebrovascular disorders. According to the WHO, more than 6 million people die annually due to cigarette smoking [1]. Medical and social economic losses due to cigarette smoking are approximately 1.8 and 2.4 trillion yen (approximately 15 and 20 billion dollars), respectively, in Japan [2], and approximately 170 and 156 billion dollars, respectively, in the US [3,4]. Therefore, the cost-effectiveness of smoking cessation treatment (SCT) for the prevention of secondary diseases is high [5], and it is recommended as part of health care cost controls. Pharmacological treatments are used as SCT, of which there are 2 types: ‘nicotine replacement therapy using nicotine preparations’ and ‘the use of the partial agonist of the nicotinic acetylcholine receptor, varenicline (VAR)’. A previous study reported that VAR is less likely to cause nicotine withdrawal symptoms and has a higher success rate than nicotine preparations [6]; therefore, it is regarded as an important drug for the promotion of non-smoking policies.

However, VAR has been implicated in the aggravation of mental diseases, such as depression, and suicide, and, as a consequence, the Food and Drug Administration (FDA) issued a warning in 2009 [7]. In Japan, the following description was added to the warning column of the package insert: It may aggravate underlying mental diseases. In the retrieval system in Japanese, CzeekV (2015/04/13 access, version 2.1.1), of the FDA voluntary adverse event report database, the Adverse Event Reporting System (FAERS), VAR is ranked in first place in the overall ranking of signals of suicidal ideation and behavior. On the other hand, a meta-analysis of randomized controlled studies showed that VAR was not associated with depression or suicide [8]. Therefore, while VAR is unlikely to cause depression and suicide, smoking cessation itself has been suggested to induce depression and suicide [9], and cigarette smokers are at a high risk of depression [10].

SSRI and SNRI, which cause few adverse reactions, are currently used as first-line drugs to treat depression [11]; however, an increase in the risk of suicide by the administration of antidepressants (ADs) was confirmed in a placebo controlled trial, and the FDA issued a warning for all SSRIs in 2003. Moreover, several studies reported AD-induced increases in suicide-related events (SRE) [12–14]. On the other hand, after a warning concerning ADs and suicide was issued in the US and Europe, the prescription rate of ADs decreased in the Netherlands with a simultaneous increase in the suicide rate [15], whereas an inverse correlation was noted between the number of prescriptions for ADs and suicide mortality [16], showing different study results due to differences in the study method.

Smoking cessation is economically important and a policy that needs to be promoted, as described above; however, the development of depression and SRE during SCT represents a major obstacle to smoking cessation.

Thus, we herein attempted to identify ADs with the lowest risk of SRE among the ADs concomitantly used in SCT with VAR from the viewpoint of the safe use of drugs by comparing the risk of SRE between VAR-treated cases with (ADs+/VAR+) and without (ADs-/VAR+) concomitant ADs treatment (Study 1). The risks associated with the administration of VAR were investigated, with consideration of the information provided by the regulatory agency that ‘VAR aggravates mental disease’. AD-treated cases were divided into those with (VAR+/ADs+) and without (VAR-/ADs+) concomitantly administered VAR and were then compared in order to examine whether VAR increases the risk of SRE (Study 2). The risk of SRE was standardized by regarding the risk as identical between ADs+/VAR+ in Study 1 and VAR+/ADs+ in Study 2, and the risk was compared between ADs-/VAR+ and VAR-/ADs+ to investigate the risk of SRE in VAR-treated cases without ADs, i.e., the latent risk of the use of VAR.

## Methods

### Study 1: Concomitant AD-associated risk of SRE in VAR-treated (SCT) cases

#### Definition of SRE

In the ICH Medical Dictionary for Regulatory Activities/Japanese version (MedDRA/J), the SMQ classification of SRE includes the following terms at the Preferred Term (PT) level: Completed suicide (MedDRA code 10010144), suicidal ideation (10042458), suicide attempt (10042464), suicidal behavior (10065604), self-injurious ideation (10051154), self-injurious behavior (10063495), depression suicidal (10012397), intentional self-injury (10022524), poisoning deliberate (10036000), and intentional overdose (10022523), and these were collectively defined as SRE.

#### Data extraction and adjustment

**Fig 1:**
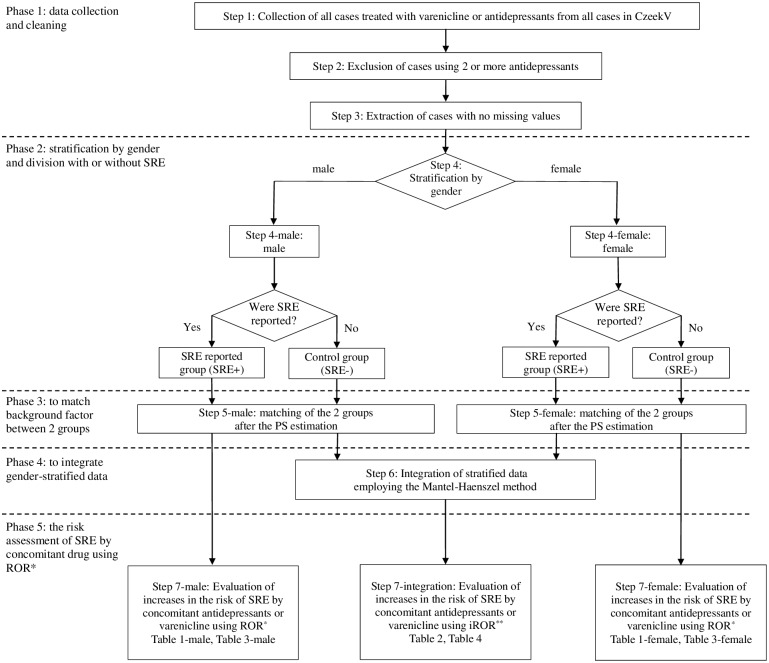
Flowchart for ROR calculations. * Reporting Odds Ratio. ** integrated Reporting Odds Ratio.

Since FAERS adopts a free-description style to enable reporting with ease, incorrect inputs and overlapping data are included. CzeekV is a unique system that organizes these data and enables searches in Japanese [17]. In the present study, all VAR-treated cases were collected from CzeekV (version 2.1.1), cases treated with 2 or more ADs were excluded, and the remaining cases were adopted as an analysis set for the case-control study. The data of this system is replaced FAERS database in Japanese. Therefore, the dataset in this study do not include any of identifying information. In order to remove selection biases as much as possible, after stratifying by gender, cases were matched based on the Propensity Score (PS) using the Statistical Package for Social Science (SPSS, version 22). In the PS estimation, 3 case background factors: age, body weight, and number of concomitantly administered drugs, were regarded as covariates, and the presence or absence of SRE was regarded as the outcome. It was not possible to apply stratification by disease severity because it was not included in case reports in FAERS. Although age was reported in many cases, the number was insufficient to analyze cases stratified by gender. Thus, only stratification by gender, a factor influencing the drug effect, was applied. A multiple logistic regression analysis employing the stepwise method was used in the PS estimation, and the PS distance (caliper value) was defined as ‘0.25 x the standard deviation of the logit transformation-applied PS estimate’. Using the PS estimate and caliper value, the SRE+ and SRE- (control) groups were matched. The standardized difference (SDD) was calculated in order to evaluate the balance of covariates of the matched data [18]. An SDD value of less than 0.1 was regarded as balanced [19]. A flow chart of the process used for reporting odds ratio (ROR) calculations is shown in Fig 1.

#### Calculation of the ROR and corresponding 95% confidence interval (ROR95%CI)

In order to investigate the risk of SRE in VAR-treated cases with concomitant ADs (ADs+/VAR+) using matched data, the ROR and ROR95%CI were calculated in each gender with the concomitant use of ADs as a factor and in the presence or absence of SRE as the outcome. In addition, the uniformity of ROR of the matched data between genders was evaluated using the Breslow-Day Test, and when ROR was uniform, the integrated ROR (iROR) and corresponding 95% CI (iROR95%CI) of ADs were calculated using the Mantel-Haenszel Test. We corrected the significance level by employing the Bonferroni method in consideration of the alpha error made by multiple comparisons [20].

### Study 2: Concomitant VAR (SCT)-associated risk of SRE in AD-treated cases

In order to investigate the risk of SRE in AD-treated cases with concomitantly administered VAR (VAR+/ADs+), AD-treated cases were collected, and those using only one AD were extracted. PS was estimated after stratification of this analysis set by gender, as described in Study 1. Cases were divided into those with and without SRE, and the 2 groups were matched. In addition, in order to investigate the influence of concomitantly administered VAR (SCT) on SRE using matched data, ROR and ROR 95% CI were calculated with the concomitant use of VAR as a factor and in the presence or absence of SRE as the outcome. The uniformity of matched data between genders was evaluated using the Breslow-Day Test, and when ROR was uniform, iROR and iROR95%CI of the AD were calculated using the Mantel-Haenszel Test.

## Results

### Study 1: Concomitant AD-associated risk of SRE in VAR-treated (SCT) cases

Changes in the number of VAR-treated cases with the flowchart in Fig 1 are shown in S1 Table. The balance of the covariates before and after matching is shown in S2 Table. The SDD values of all covariates after matching were less than 0.1, which was close to randomization.

Using matched data, ROR and ROR 95% CI were calculated with the presence or absence of concomitantly administered ADs in VAR-treated cases as a factor and in the presence or absence of SRE as an outcome. The results obtained are summarized in Table 1 (see the minimal dataset in S3 Table).

**Table 1:** Influence of concomitantly administered ADs on the risk of SRE in VAR-treated cases.

**Male**							
	**VAR-treated cases**						
	**ADs+/VAR+**		**ADs-/VAR+**				
	**SRE+**	**SRE-**	**SRE+**	**SRE-**	**ROR**	**ROR 95%CI**	**p-value**
All AD-treated cases	58	30	601	629	2.02	1.28–3.19	0.002*
By drug							
Mirtazapine	7	1	601	629	7.33	0.90–59.73	0.063
Citalopram	12	2	601	629	6.28	1.40–28.18	0.016
Bupropion	9	4	601	629	2.35	0.72–7.69	0.157
Duloxetine	6	3	601	629	2.09	0.52–8.41	0.299
Fluoxetine	5	3	601	629	1.74	0.42–7.33	0.45
Venlafaxine	5	3	601	629	1.74	0.42–7.33	0.45
Sertraline	9	6	601	629	1.57	0.56–4.44	0.395
Amitriptyline	2	2	601	629	1.05	0.15–7.45	0.961
Paroxetine	3	3	601	629	1.05	0.21–5.21	0.952
**Female**							
	**VAR-treated cases**						
	**ADs+/VAR+**		**ADs-/VAR+**				
	**SRE+**	**SRE-**	**SRE+**	**SRE-**	**ROR**	**ROR 95%CI**	**p-value**
All AD-treated cases	163	86	746	823	2.09	1.58–2.76	< 0.001*
By drug							
Mirtazapine	6	1	746	823	6.62	0.80–55.11	0.08
Sertraline	19	6	746	823	3.49	1.39–8.79	0.008
Duloxetine	21	7	746	823	3.31	1.40–7.83	0.006
Bupropion	15	6	746	823	2.76	1.06–7.15	0.037
Fluoxetine	27	14	746	823	2.13	1.11–4.09	0.023
Venlafaxine	25	13	746	823	2.12	1.08–4.18	0.03
Trazodone	6	4	746	823	1.65	0.47–5.89	0.439
Citalopram	12	13	746	823	1.02	0.46–2.25	0.961
Amitriptyline	5	11	746	823	0.5	0.17–1.45	0.201
Paroxetine	21	0	746	823	Inf	5.71—Inf	not calculated
Nortriptyline	3	0	746	823	Inf	0.45—Inf	not calculated
Amoxapine	1	0	746	823	Inf	0.03 -Inf	not calculated
Clomipramine	1	0	746	823	Inf	0.03 -Inf	not calculated
Trimipramine	1	0	746	823	Inf	0.03 -Inf	not calculated
Lofepramine	1	0	746	823	Inf	0.03 -Inf	not calculated
Imipramine	0	1	746	823	0	0.00—Inf	not calculated

Inf, infinite.

* significantly different after Bonferroni’s correction for multiple comparisons.

In male cases (Table 1-male), concomitantly administered ADs significantly increased the risk of SRE (ROR 2.02, ROR95%CI 1.28–3.19). Nine types of ADs were extracted, as shown in Table 1-male. None of the antidepressants tested significantly increased the risk of SRE after the correction of the significance level by the Bonferroni method.

Concomitantly administered ADs also significantly increased the risk of SRE in female cases (ROR 2.09, ROR95%CI 1.58–2.76) (Table 1-female). Sixteen types of ADs were extracted, as shown in Table 1-female. None of the antidepressants tested significantly increased the risk of SRE after the correction of the significance level by the Bonferroni method.

The results of the ROR uniformity test (Breslow-Day Test) and iROR calculation (Mantel-Haenszel Test) are shown in Table 2. As shown by values marked with ^a^ in Table 2, the ROR of citalopram was not uniform between the genders. In the other ADs, ROR was uniform and it was possible to calculate iROR. As shown in Table 2, concomitantly administered ADs significantly increased the risk of SRE (iROR 2.07, iROR95%CI 1.63–2.63), while only duloxetine (ROR 2.94, ROR95%CI 1.41–6.10) significantly increased the risk of SRE. iROR was less than 1.00 in amitriptyline-treated cases only, but was not significant. Since there was no reported male case of concomitantly administered nortriptyline, amoxapine, clomipramine, trimipramine, lofepramine, or imipramine, it was impossible to investigate uniformity.

**Table 2:** Uniformity test of ROR (Breslow-Day Test) and calculation of integrated ROR (Mantel-Haenszel Test) in VAR-treated cases.

	Breslow-Day Test			Mantel-Haenszel Test		
	χ^2^-value	Df	p-value	iROR	iROR95%CI	p-value
All AD-treated cases	0.015	1.00	0.904	2.07	1.63–2.63	< 0.001*
By drug						
Mirtazapine	0.004	1.00	0.947	6.98	1.57–30.99	0.007
Duloxetine	0.304	1.00	0.581	2.94	1.41–6.10	0.004*
Bupropion	0.042	1.00	0.838	2.59	1.24–5.45	0.015
Sertraline	1.296	1.00	0.255	2.52	1.26–5.02	0.009
Fluoxetine	0.061	1.00	0.805	2.06	1.14–3.73	0.022
Venlafaxine	0.059	1.00	0.809	2.05	1.11–3.78	0.028
Trazodone	0.194	1.00	0.660	1.39	0.51–3.76	0.518
Amitriptyline	0.427	1.00	0.513	0.59	0.23–1.50	0.360
Citalopram	5.010	1.00	0.025^a^	#	#	not calculated

df, degree of freedom.

^a^ non-uniformity.

^#^ Value could not be calculated due to the non-uniformity of ROR.

* significantly different after Bonferroni’s correction for multiple comparisons.

### Study 2: Concomitant VAR (SCT)-associated risk of SRE in AD-treated cases

Changes in the number of AD-treated cases with the flowchart in Fig 1 are shown in S1 Table. The 8 types of ADs, the ROR of which were recognized as uniform in Table 2, were analyzed in Study 2. Cases treated with these 8 drugs were stratified by gender and 1-to-1 matched. The results obtained are shown in S2 Table. Using matched data, ROR and ROR 95% CI were calculated in the presence or absence of concomitantly administered VAR in AD-treated cases as a factor and in the presence or absence of SRE as an outcome. The results obtained are summarized in Table 3.

**Table 3:** Influence of concomitantly administered VAR on the risk of SRE in AD-treated cases.

**Male**							
	**AD-treated cases**						
	**VAR+/ADs+**		**VAR-/ADs+**				
	**SRE+**	**SRE-**	**SRE+**	**SRE-**	**ROR**	**ROR 95%CI**	**p-value**
All AD-treated cases	56	9	1574	1621	6.37	3.14–12.90	< 0.001*
Bupropion	8	1	178	185	8.31	1.03–67.16	0.047
Venlafaxine	5	1	282	286	5.07	0.59–43.68	0.14
Sertraline	8	2	372	378	4.06	0.86–19.27	0.078
Fluoxetine	10	3	236	243	3.43	0.93–12.62	0.064
Trazodone	1	1	19	19	1	0.01–82.52	1.000
Mirtazapine	8	0	49	57	Inf	1.86—Inf	not calculated
Duloxetine	6	0	184	190	Inf	1.19—Inf	not calculated
Amitriptyline	1	0	80	81	Inf	0.03—Inf	not calculated
**Female**							
	**AD-treated cases**						
	**VAR+/ADs+**		**VAR-/ADs+**				
	**SRE+**	**SRE-**	**SRE+**	**SRE-**	**ROR**	**ROR 95%CI**	**p-value**
All AD-treated cases	174	21	2884	3037	8.23	5.53–13.76	< 0.001*
Sertraline	24	1	456	479	25.21	3.40–187.13	0.002*
Fluoxetine	32	4	364	392	8.62	3.02–24.60	< 0.001*
Venlafaxine	28	4	483	507	7.35	2.56–21.10	< 0.001*
Trazodone	6	1	52	57	6.58	0.77–56.47	0.086
Amitriptyline	5	1	86	90	5.23	0.60–45.71	0.135
Bupropion	15	5	216	226	3.14	1.12–8.79	0.029
Duloxetine	23	0	383	406	Inf	6.03—Inf	not calculated
Mirtazapine	6	0	60	66	Inf	1.23—Inf	not calculated

Inf, infinite.

* significantly different after Bonferroni’s correction for multiple comparisons.

In male cases (Table 3-male), when the type of AD was not specified, concomitantly administered VAR significantly increased the risk of SRE (ROR 6.37, ROR95%CI 3.14–12.90). Regarding ADs, concomitantly administered VAR did not significantly increase the risk of SRE in cases treated with any of the antidepressants tested after the correction of the significance level (see the minimal dataset in S4 Table).

Similarly, in female cases (Table 3-female), when the type of AD was not specified, concomitantly administered VAR significantly increased the risk of SRE (ROR 8.23, ROR95%CI 5.53–13.76). Regarding ADs, after the correction of the significance level, concomitantly administered VAR significantly increased the risk of SRE in cases treated with sertraline (ROR 25.21, ROR95%CI 3.40–187.13), fluoxetine (ROR 8.62, ROR95%CI 3.02–24.60), and venlafaxine (ROR 7.35, ROR95%CI 2.56–21.10) (see the minimal dataset in S4 Table).

The results of the ROR uniformity test (Breslow-Day Test) and iROR calculation (Mantel-Haenszel Test) for each of the 8 ADs are shown in Table 4. Based on Table 4, when the type of AD was not specified, concomitantly administered VAR significantly increased the risk of SRE (iROR 8.02, iROR95%CI 5.47–11.76). Regarding ADs, concomitantly administered VAR significantly increased the SRE risk in cases treated with sertraline (iROR 10.97, iROR95%CI 3.21–37.51), venlafaxine (iROR 6.88, iROR95%CI 2.67–17.74), fluoxetine (iROR 6.34, iROR95%CI 2.80–14.35), and bupropion (iROR 4.02, iROR95%CI 1.60–10.12). Calculations were not possible for amitriptyline, mirtazapine, or duloxetine because complete data were not available.

**Table 4:** Uniformity test of ROR (Breslow-Day Test) and calculation of integrated ROR (Mantel-Haenszel Test) in AD-treated cases.

	Breslow-Day Test			Mantel-Haenszel Test		
	χ^2^-value	Df	p-value	iROR	iROR95%CI	p-value
All AD-treated cases	0.520	1.00	0.471	8.02	5.47–11.76	< 0.001*
Sertraline	2.397	1.00	0.122	10.97	3.21–37.51	< 0.001*
Venlafaxine	0.093	1.00	0.761	6.88	2.67–17.74	< 0.001*
Fluoxetine	1.210	1.00	0.271	6.34	2.80–14.35	< 0.001*
Bupropion	0.708	1.00	0.400	4.02	1.60–10.12	0.002*
Trazodone	1.204	1.00	0.273	3.71	0.67–20.61	0.172
Amitriptyline	0.189	1.00	0.664	6.29	#	not calculated
Mirtazapine	#	#	#	#	#	not calculated
Duloxetine	#	#	#	#	#	not calculated

df, degree of freedom.

^#^ Calculations were not possible because no case was reported.

* significantly different after Bonferroni’s correction for multiple comparisons.

## Discussion

### Study 1: Concomitant AD-associated risk of SRE in VAR-treated (SCT) cases

In male and female cases, none of the antidepressants tested significantly increased the risk of SRE (Table 1). However, when the genders were combined, concomitant duloxetine significantly increased the risk of SRE over that in the control group (Table 2). Furthermore, although a significant difference was not detected, iROR, i.e., the risk of SRE, varied among the antidepressants concomitantly administered to VAR-treated cases. For example, the risk of amitriptyline-induced SRE was the lowest among the antidepressants tested, but was not significant (ROR = 0.59, ROR95%CI 0.23–1.50). In addition, iROR was less than 1.00 in amitriptyline-treated cases only, suggesting that the concomitant use of amitriptyline reduces the risk of SRE more that in the control group. The number of citalopram-treated cases was high in both genders, and ROR was not uniform in the Breslow-Day Test in citalopram-treated cases only. Thus, the risk of SRE differed between the genders (Table 1). However, the absence of a gender difference has been reported in the drug effects of citalopram [21], suggesting that this result was incidental.

Since Carol et al. [22] reported that the hazard ratio for AD-associated completed suicide was significantly low in amitriptyline-treated cases only, amitriptyline may have SRE risk-reducing effects. Amitriptyline is more frequently used at specialized medical institutions, and the lower risk of SRE in amitriptyline-treated cases may have been due to the management of depressive symptoms and adverse effects at these medical institutions.

### Study 2: The risk of SRE associated with the concomitant administration of VAR (SCT) in AD-treated cases

It was not possible to calculate iROR95%CI for amitriptyline, mirtazapine, or duloxetine. Concomitantly administered VAR may have increased the risk of SRE, regardless of the type of AD, over that in the control group, showing that SCT with VAR is likely to increase the risk of depression-associated suicide. Amitriptyline decreased the risk of SRE in Study 1, but not in Study 2.

### Potential of SCT as a latent factor of SRE

**Fig 2:**
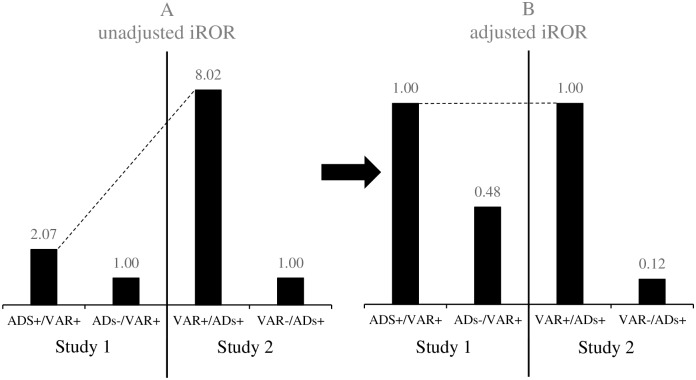
(A) Unadjusted iROR and (B) adjusted iROR of varenicline (VAR) and antidepressants (ADs) determined by matching in each study.

The iROR of integrated male and female data in Studies 1 and 2 is shown in Fig 2A. iROR was high in Study 2, indicating that the concomitant VAR-associated risk in AD-treated cases (Study 2) was higher than the concomitant AD-associated risk in VAR-treated cases (Study 1). However, this comparison was not possible because the background of the control group differed between Studies 1 and 2 (ADs-/VAR+ and VAR-/ADs+, respectively). Thus, assuming that the case groups common to both studies, VAR+/ADs+ and ADs+/VAR+, were similar populations, the iROR of these groups were regarded as 1.00 for the baseline in adjustments. The results obtained are shown in Fig 2B. VAR-/ADs+ and ADs-/VAR+ were compared in Fig 2B. The bar graph of ADs-/VAR+ was higher than that of VAR-/ADs+, suggesting that SCT with VAR is a latent factor increasing the risk of SRE more than that by ADs used to treated depression.

SCT with VAR has been suggested to increase the risk of SRE. However, it currently remains unclear whether VAR itself has a suicide-inducing effect or SCT induces suicide in smokers already at risk of suicide. Factors inducing suicide include ‘issues with patients themselves, such as SCT with VAR and mental disorders’ and ‘the social environment of patients, such as stigma from a patient’s family and medical health professionals’ [23]. An accurate evaluation of the suicide-inducing risk of the latter is difficult because cases cannot be collected from those reported to FAERS. However, as shown in Fig 2B, the risk of SRE was higher in ADs-/VAR+, in which stigma was not an inducer of SRE, than in VAR-/ADs+, and this did not overestimate the risk of SRE associated with the administration of VAR.

### Limitations

Data from FAERS were analyzed, but only a small number of case reports included gender, the 3 covariates, and adverse events because FAERS adopts the free-description style, and there were many missing data. For example, out of 57,440 VAR-treated cases that were regarded as an analysis set in Study 1, all covariates were reported in 23,008 cases only, and, thus, approximately 60% were not included in the PS estimation. It is possible that an accurate ROR may not have been calculated.

Since case reports in FAERS are cross-sectional, the order of timing of taking drugs and the development of several diseases in a case is currently unclear. Therefore, cases that started smoking cessation during the treatment of depression may have been included among cases that developed depression during SCT, thereby influencing the ROR estimation.

Since the number of covariates is ideally 1/10-1/7 of the number of cases in PS estimations, the number of covariates was insufficient in the present study. When PS is estimated and matched in analyses with a small number of covariates, it is possible that cases with close PS, but a different balance of covariates were matched.

## Conclusion

The treatment of depression with ADs during SCT with VAR is more likely to increase the risk of SRE more than that with the treatment of depression with ADs (control group). SCT with VAR has been suggested as a latent factor inducing depression and SRE, and the risk associated with this treatment is stronger than that associated with the treatment of depression with ADs.

When depression develops during SCT, the risk of SRE increases, for which concomitant amitriptyline may decrease the risk of suicide. In contrast, concomitant mirtazapine may increase the risk of SRE because its iROR was the highest.

## Supporting Information

S1 Table Changes in the number of cases treated with each drug in steps used for ROR calculations. (XLSX)

S2 Table Balance of covariates by standardized difference (SDD) in VAR-treated and AD-treated cases. (XLSX)

S3 Table The minimal dataset underlying results obtained in Study 1. Report No. is the report number in CzeekV, NCD stands for the number of concomitant drugs, and SRE represents whether SRE were reported.
(XLSX)

S4 Table The minimal dataset underlying results obtained in Study 2. Report No. is the report number in CzeekV, NCD stands for the number of concomitant drugs, SRE represents whether SRE were reported, and VAR represents whether VAR was used.
(XLSX)

## References

[1] World Health Organization [internet]. Tobacco [updated 2015 July; cited 2015 July 15]. Available: http://www.who.int/mediacentre/factsheets/fs339/en/.

[2] Institute for Economics and Policy [internet]. A study on the role of non-smoking policy [updated 2010; cited 2015 July 10]. Available: http://www.ihep.jp/publications/report/search.php?dl=26&i=1.

[3] SURGEON GENERAL.GOV [internet]. The Health Consequences of Smoking-50Years of Progress: A Report of the Surgeon General, 2014 [updated 2014; cited 2015 July 23]. Available: http://www.surgeongeneral.gov/library/reports/50-years-of-progress/index.html.

[4] Xu Xin, Bishop Ellen E., Kennedy Sara M., Simpson Sean A., Pechacek Terry F. Annual Healthcare Spending Attributable to Cigarette Smoking: An Update. Am J Prev Med. 2015; 48(3):326–333. 10.1016/j.amepre.2014.10.012 25498551 PMC4603661

[5] Fiore Michael C., Jaen Carlos Roberto, Baker Timothy B., Bailey William C., Benowitz Neal L., Curry Susan J., et al Treating Tobacco Use and Dependence: 2008 Update, clinical practice guideline. U.S. Department of Health and Human Services.

[6] Aubin H-J, Bobak A, Britton J R, Oncken C, Billing C B Jr, Gong J, et al Varenicline versus transdermal nicotine patch for smoking cessation: result from a randomized open-label trial. Thorax. 2008; 63:717–724. 10.1136/thx.2007.090647 18263663 PMC2569194

[7] Food U.S. and Drug Administration [internet]. FDA Requires New Boxed Warnings for the Smoking Cessation Drugs Chantix and Zyban [updated 2013 Aug 16; cited 2015 July 25]. Available: http://www.fda.gov/Drugs/DrugSafety/DrugSafetyPodcasts/ucm170906.htm.

[8] Thomas Kyla H, Martin Richard M, Knipe Duleeka W, Higgins Julian P T, Gunnell David. Risk of neuropsychiatric adverse events associated with varenicline: systematic review and meta-analysis. BMJ. 2015; 350: h1109 10.1136/bmj.h1109 25767129 PMC4357491

[9] Breslau Naomi, Kilbey M. Marlyne, Andreski Patricia. Nicotine dependence and major depression. New evidence from a prospective investigation. Arch Gen Psychiatry. 1993;50(1):31–35 8422219 10.1001/archpsyc.1993.01820130033006

[10] Zvolensky Michael J., Bakhshaie Jafar, Sheffer Christine, Perez Adriana, Goodwin Renee D. Major depressive disorder and smoking relapse among adults in the United States: A 10-year, prospective investigation. Psychiatry Res. 2015;226(1):73–77. 10.1016/j.psychres.2014.11.064 25650047 PMC4448723

[11] Health & Social Care Information Centre [internet]. Prescription Cost Analysis, England– 2014 [publication date 2015 Apr 08; cited 2015 July 15]. Available: http://www.hscic.gov.uk/catalogue/PUB17274.

[12] Jick Hershel, Kaye James A., Jick Susan S. Antidepressants and the risk of suicidal behaviors. JAMA. 2004; 292(3):338–343 15265848 10.1001/jama.292.3.338

[13] Martinez Carlos, Rietbrock Stephan, Wise Lesley, Ashby Deborah, Chick Jonathan, Moseley Jane, et al Antidepressant treatment and the risk of fatal and non-fatal self harm in first episode depression: nested case—control study. BMJ. 2005; 330(7488): 389 15718538 10.1136/bmj.330.7488.389 PMC549107

[14] Stone Marc, Laughren Thomas, Jones M Lisa, Levenson Mark, Holland P Chris, Hughes Alice, et al Risk of suicidality In clinical trials of antidepressants in adults: analysis of proprietary data submitted to US Food and Drug Administration. BMJ. 2009; 339:b2880 10.1136/bmj.b2880 19671933 PMC2725270

[15] Gibbons Robert D., Brown C. Hendricks, Hur Kwan, Marcus Sue M., Bhaumik Dulal K., Erkens Joëlle A., et al Early evidence on the effects of regulators’ suicidality warning on SSRI prescriptions and suicide in children and adolescents. Am J Psychiatry. 2007; 164(9):1356–63 17728420 10.1176/appi.ajp.2007.07030454

[16] Nakagawa Atsuo, Grunebaum Michael F., Ellis Steven P., Oquendo Maria A., Kashima Haruo, Gibbons Robert D., et al Association of suicide and antidepressant prescription rates in japan, 1999–2003. J Clin Psychiatry. 2007; 68(6): 908–916 17592916 10.4088/jcp.v68n0613 PMC3804897

[17] Sakaeda Toshiyuki, Tamon Akiko, Kadoyama Kaori, Okuno Yasushi. Data Mining of Public Version of the FDA Adverse Event Reporting System. Int J Med Sci. 2013; 10(7):796–803. 10.7150/ijms.6048 23794943 PMC3689877

[18] Yang Dongsheng, Dalton Jarrod E. Standardized Difference: An Index to Measure the Effect Size between Two Groups. SAS Global Forum 2012.

[19] Wang Yongji, Cai Hongwei, Li Chanjuan, Jiang Zhiwei, Wang Ling, Song Jiugang, et al Optimal caliper width for propensity score matching of three treatment groups: a Monte Carlo study. PLoS One. 2013; 8(12):e81045 10.1371/journal.pone.0081045 24349029 PMC3859481

[20] Bland J Martin, Altman Douglas G. Multiple significance tests: the Bonferroni method. BMJ. 1995; 310: 170 7833759 10.1136/bmj.310.6973.170 PMC2548561

[21] Silverstein Brett, Patel Priya. Poor Response to Antidepressant Medication of Patients with Depression Accompanied by Somatic Symptomatology in the STAR*D Study. Psychiatry Res. 2011; 187(1–2):121–4. 10.1016/j.psychres.2010.12.026 21216475

[22] Coupland Carol, Hill Trevor, Morriss Richard, Arthur Antony, Moore Michael, Hippisley-Cox Julia. Antidepressant use and risk of suicide and attempted suicide ROR self harm in people aged 20 to 64: cohort study using a primary care database. BMJ. 2015; 350: h517 10.1136/bmj.h517 25693810 PMC4353276

[23] Pompili M., Mancinelli I., Tatarelli R. Stigma as a cause of suicide. Br J Psychiatry. 2003; 183: 173–174. 12893678

